# First principle investigation of the linker length effects on the thermodynamics of divalent pseudorotaxanes

**DOI:** 10.3762/bjoc.11.78

**Published:** 2015-05-08

**Authors:** Andreas J Achazi, Doreen Mollenhauer, Beate Paulus

**Affiliations:** 1 Institut für Chemie und Biochemie, Freie Universität Berlin, Takustr. 3, 14195 Berlin, Germany; 2 Physikalisch-Chemisches Institut, Justus-Liebig-Universität Gießen, Heinrich-Buff-Ring 58, 35392 Gießen, Germany

**Keywords:** density functional theory (DFT), dispersion correction, Gibbs energy, pseudorotaxanes, solvent effects, COSMO-RS

## Abstract

The Gibbs energies of association (Gibbs free (binding) energies) for divalent crown-8/ammonium pseudorotaxanes are determined by investigating the influence of different linkers onto the binding. Calculations are performed with density functional theory including dispersion corrections. The translational, rotational and vibrational contributions are taken into account and solvation effects including counter ions are investigated by applying the COSMO-RS method, which is based on a continuum solvation model. The calculated energies agree well with the experimentally determined ones. The shortest investigated linker shows an enhanced binding strength due to electronic effects, namely the dispersion interaction between the linkers from the guest and the host. For the longer linkers this ideal packing is not possible due to steric hindrance.

## Introduction

**Figure 1:**
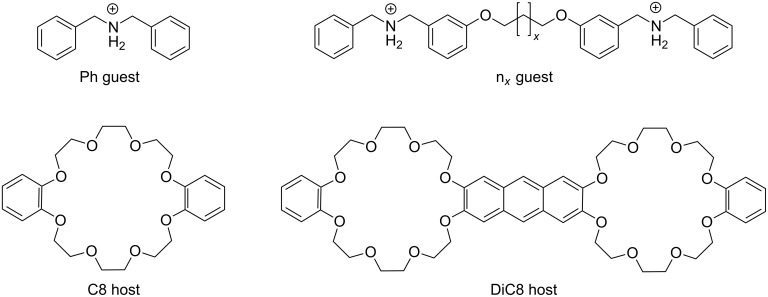
Structures of the mono- and divalent guest and host molecules. The linker in the divalent guest molecule is varied with *x* = 0, 1 or 2.

If two or more binding sites of a molecular system are involved in the association process, the interaction energy can be significantly increased compared to the sum of the individual binding energies. This effect is called multivalency [1] and is mainly observed in biochemical systems [2–9]. But the concept of multivalency can be transferred to supramolecular assemblies with suitable building blocks [10–12] including (pseudo)rotaxanes [13–15] as well. One common building block for pseudorotaxanes is the crown/ammonium binding motif. In this motif ammonium can bind on top of small crown ethers, e.g., crown-6, or can pass through larger crown ethers, e.g., crown-8. Jiang et al. [16] have investigated the assembly thermodynamics and kinetics of divalent crown-8/ammonium pseudorotaxanes with different linkers. The shortest linker shows a much larger chelate cooperativity than the longer linkers due to non-innocent linkers that contribute to the binding. To analyze the individual contributions to the binding, we perform first principle calculations of the model system shown in Figure 1, which is strongly related to the experimentally investigated systems of Jiang et al. [16]. The only difference is that 1,4-diazanaphthalene groups of the host molecule are replaced by phenyl groups and the side chains of the anthracene bridge in the divalent host are neglected. In addition to the electronic contributions, enthalpic and entropic temperature effects as well as solvent effects are included in our simulations in order to compare to experimentally obtained Gibbs energy of association.

## Results and Discussion

In order to investigate the cooperativity effects of the binding between divalent host molecules and divalent guest molecules it is important to firstly describe the monovalent binding motif computationally as accurately as possible and to understand the underlying effects that contribute to the binding. Three major terms have to be considered in the evaluation of the Gibbs energy of association Δ*G* to model the reaction in solution at finite temperature with reasonable accuracy. 1) The electronic association energy Δ*E* is calculated [17] with the DFT functional TPSS-D3(BJ) [18–20] and the basis set def2-TZVP [21–22]. A comparison with the electronic association energy determined with the DF-LCCSD(T) method [23–24] at the extrapolated basis set limit shows good agreement (see Table 1). Already the DF-LCCSD(T) with the cc-pVTZ basis set deviates only by 5% from the TPSS-D3(BJ) value, whereas the basis set extrapolated value is more or less equivalent to the TPSS-D3(BJ) value (deviation less than 0.3%). This very good agreement is somewhat fortunate, because a basis set extrapolation with DZ and TZ is only accurate to within a few percent. Additionally, the possible errors of the functional and the dispersion correction can also be in the range of 10% for the system under investigation. A more detailed analysis of the accuracy of the TPSS-D3(BJ) functional has been performed for the crown-6/ammonium complex in [25]. Another point to remark is that even for the monovalent system about 36% of the electronic interaction energy is due to the dispersion correction. 2) The finite temperature effects from translation, rotation and vibration are calculated with an approach from Grimme [26], which partially treats the low-lying vibrations as hindered rotations (TPSS-D3(BJ)/def2-SVP [22,27] for vibrations). 3) The influence of the solvent for the association process in solution is derived from the difference of the solvation effects of the product and the reagents, calculated with COSMO-RS [28–29]. For the COSMO-RS (BP_TZVP_C30_1301.ctd parameterization) calculation all structures have been optimized in an ideal conductor [30] and in vacuum with BP86/def-TZVP [31–34]. This procedure yields very good results for the Gibbs energy of association in the case of the crown-6/ammonium complex in comparison with experiment [25]. For the simulations of the crown-8/ammonium systems the same solvent as in the experiment [16] is used, namely a 2.2:1 mixture of chloroform/acetonitrile. The influence of the counter ion PF_6_^−^ onto the Gibbs energy of association is taken into account explicitly.

**Table 1:** Electronic association energy Δ*E* for Ph@C8*.^a^

system	method	Δ*E* (kJ/mol)

Ph@C8*	TPSS/def2-TZVP	−134.9
Ph@C8*	TPSS-D3(BJ)/def2-TZVP	−210.5
Ph@C8*	DF-LCCSD(T)/cbs(DZ-TZ)	−210.0
Ph@C8*	DF-LCCSD(T)/cc-pVDZ	−174.7
Ph@C8*	DF-LCCSD(T)/cc-pVTZ	−199.9

^a^Δ*E* calculated at TPSS-D3(BJ)/def2-TZVP level of theory is not identical to the one in Table 2, because there another conformer (a slightly more stable one) is used. The Ph@C8* structure has been optimized with TPSS-D3(BJ)/def2-TZVP. For the other methods only single point calculations are done.

**Figure 2:**
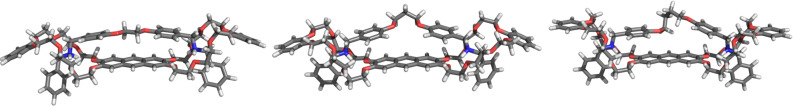
Optimized gas phase structures (TPSS-D3(BJ)/def2-TZVP) of the divalent complexes n_0_@DiC8, n_1_@DiC8 and n_2_@DiC8.

The divalent host molecules consist of two crown-8 ethers that are linked by an anthracene bridge. For the divalent guest molecule different flexible linkers, namely –O(CH_2_)_2_O– (n_0_), –O(CH_2_)_3_O– (n_1_) and –O(CH_2_)_4_O– (n_2_) have been investigated both experimentally in [16] and computationally. The results for the electronic association energy Δ*E*, the Gibbs energy of association Δ*G* in the gas phase and its enthalpic (Δ*H*) and entropic (−*T*Δ*S*) contributions are given in Table 2. Comparing the electronic association energy for the n_0_ guest in the divalent case with the doubled value of the monovalent (Ph@C8) system, an electronic cooperativity effect of 9.7 kJ/mol is discovered. When the linker length is increased, this electronic cooperativity effect is lost, and a lower electronic association energy (by 11.3 kJ/mol) is discovered for the divalent system with the n_1_ linker compared to two monovalent systems. For the longer n_2_ linker the electronic association energy is even lower by 24.2 kJ/mol for the divalent system compared to two monovalent systems. This is mainly due to the dispersive interaction of the linking unit (two phenyl rings and the linker), which in case of the n_0_ guest fits perfectly on top of the anthracene linker of the DiC8 host. The distance between the linker of the host and the linker of the n_0_ guest is around 3.7 Å, quite close to an ideal distance for the π–π stacking of two benzene rings. The n_1_ and n_2_ guest do not perfectly fit with the host (Figure 2). In the n_1_-case the linker is folded away from the anthracene bridge, and for the n_2_-case one phenyl ring is twisted away due to steric constraints.

**Table 2:** Electronic association energy Δ*E* and Gibbs energy of association Δ*G* in the gas phase at room temperature (*T* = 298.15 K).^a^

system	Δ*E* (kJ/mol)	Δ*G* (kJ/mol)	Δ*H* (kJ/mol)	−TΔ*S* (kJ/mol)

Ph@C8	−215.6	−130.2	−204.8 (+10.9)	+74.6
n_0_@DiC8	−440.9	−339.3	−422.6 (+18.3)	+83.3
n_1_@DiC8	−419.9	−317.5	−402.6 (+17.3)	+85.2
n_2_@DiC8	−407.0	−299.8	−386.8 (+20.2)	+87.0

^a^The enthalpic (Δ*H*) and entropic (−*T*Δ*S*) contribution to Δ*G* are given. The Δ*H* contribution resulting from finite temperatures is given in brackets.

The Gibbs energy of association Δ*G* in the gas phase of the divalent systems (Table 2) result in the same trend as observed for the electronic association energy Δ*E*, because the enthalpic (Δ*H*) and entropic (−*T*Δ*S*) contributions are similar for n_0_@DiC8, n_1_@DiC8 and n_2_@DiC8.

In Table 3, the Gibbs energies of association in solution with and without counter ion are compared to the calculated electronic association energies, Gibbs energies of association in the gas phase and to the measured experimental values. For the monovalently bound system Ph@C8 the computationally obtained value of Δ*G* (−12.6 kJ/mol) agrees well with the experimentally determined value (−15.0 kJ/mol). The Gibbs energies of association in gas phase and the Gibbs energies of association in solution show similar differences between n_0_@DiC8, n_1_@DiC8 and n_2_@DiC8 as the electronic association energies. Hence, the dependence on the linker length is of electronic origin and not affected by temperature or solvent effects. Including the counter ion in the determination of Δ*G* has a much weaker effect in the divalent case compared to the monovalent one, because the guest molecule is larger and the positive charge of the amide group can be distributed better over the molecule. For the divalent pseudorotaxanes the absolute agreement between the calculated and the experimentally determined Gibbs energies is not as good as in the case of monovalent binding, but the same trends are observed in the simulations as in experiment. The divalent pseudorotaxane with the n_0_ linker shows a significantly stronger binding than the longer molecules.

**Table 3:** Gibbs energy of association Δ*G* in solution.^a^

system	Δ*E* (kJ/mol)	Δ*G* gas phase (kJ/mol)	Δ*G* solution (kJ/mol)	Δ*G* counter ion (kJ/mol)	ΔG experiment (kJ/mol)

Ph@C8	−215.6	−130.2	−1.1	−12.6	−15.0
n_0_@DiC8	−440.9	−339.3	−42.5	−44.3	−25.1
n_1_@DiC8	−419.9	−317.5	−24.2	−28.9	−17.4
n_2_@DiC8	−407.0	−299.8	−11.5	−15.3	−16.2

^a^Electronic association energy Δ*E*, Gibbs energy of association Δ*G* in gas phase and in solution, in the latter case with and without inclusion of the counter ion PF_6_^−^, and experimentally determined Δ*G* for monovalent and divalent pseudorotaxanes in a 2.2:1 solvent mixture of chloroform/acetonitrile at room temperature (*T* = 298.15 K) are presented.

**Figure 3:**
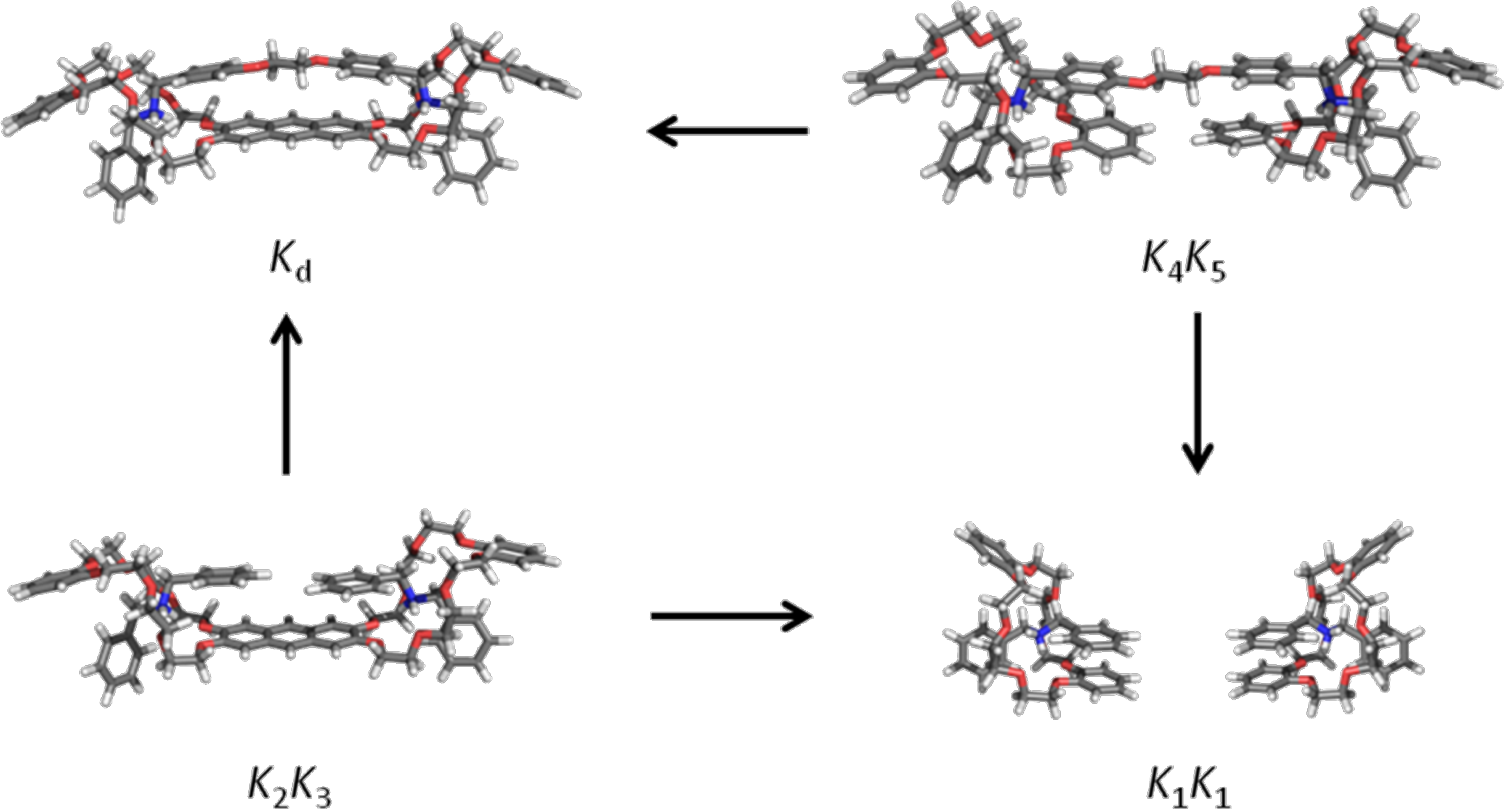
Double mutant cycle for n_0_@DiC8. The *K* variables are declared in Table 4 and are used in Equation 1. Top left: n_0_@DiC8, top right: n_0_@2C8, bottom left: 2Ph@DiC8 and bottom right: two Ph@C8. The figures show the optimized gas phase structures.

Additionally, the full double mutant cycle from [16] has been calculated (Figure 3 and Table 4). The Gibbs energy of association Δ*G* in case of Ph@DiC8 and n_0_@2C8 is in good agreement with the experimental data. For 2Ph@DiC8 and n_0_@C8 the deviation is larger just as for the divalent systems in Table 3. This deviation strongly affects the calculated equilibrium constants *K*, because Δ*G* is included exponentially in *K*. Therefore only a qualitatively discussion of the equilibrium constants is possible. With the determined equilibrium constants *K*, the effective molarity EM can be calculated [16]:

[1]
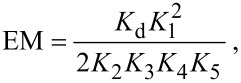


[2]
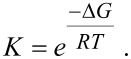


According to Hunter and Anderson [35] EM·*K*_1_ can be used to quantify cooperativity. If EM·*K*_1_ ≈ 1, the system shows no or small cooperativity, if EM·*K*_1_ >> 1 the systems shows positive cooperativity and for EM·*K*_1_ << 1 the opposite occurs. The data for the EM·*K*_1_ values are all based on the double mutant cycle of n_0_, because the experimental data are also using only the double mutant cycle of n_0_ for n_1_ and n_2_. The experiment shows that n_0_@DiC8 (EM·*K*_1_(exp.) = 55.3) has a highly positive cooperativity while n_1_@DiC8 (EM·*K*_1_(exp.) = 2.4) and n_2_@DiC8 (EM·*K*_1_(exp.) =1.5) have no significant cooperativity. In contrast to the experiment, the calculations show that n_0_@DiC8 (EM·*K*_1_(cal.) = 1.6·10^8^), n_1_@DiC8 (EM·*K*_1_(cal.) = 3.1·10^5^) and n_2_@DiC8 (EM·*K*_1_(cal.) = 1.3·10^3^) have highly positive cooperativity, but all calculated values are much too high compared to experiment due to the deviations of Δ*G* for 2Ph@DiC8 and n_0_@C8. Despite these errors the calculation shows in agreement to experiment, that n_0_@DiC8 has a much higher EM·*K*_1_ value than n_1_@DiC8 and n_2_@DiC8. So the calculations confirm that the linkers contribute to the binding strength in the divalent pseudorotaxanes and can be called non-innocent as in [16].

**Table 4:** Gibbs energy of association ΔG in solution (2.2:1 chloroform/acetonitrile, 298.15 K) and equilibrium constant K for the systems from the double mutant cycle.^a^

system	Δ*G* counter ion (kJ/mol)	*K* (mol^−1^·L^−1^)	#*K*	Δ*G* experimental (kJ/mol)	*K* experimental (mol^−1^·L^−1^)

Ph@C8	−12.6	161.2	*K*_1_	−15.0	420
Ph@DiC8	−16.2	677.8	*K*_2_	−16.4	735
2Ph@DiC8	−5.11	7.9	*K*_3_	−12.3	145
n_0_@C8	+1.4	0.6	*K*_4_	−16.3	714
n_0_@2C8	−13.8	261.6	*K*_5_	−13.3	220
n_0_@DiC8	−44.3	57679927.3	*K*_d_	−25.1	25000
n_1_@DiC8	−28.9	115627.5	*K*_d_	−17.4	1100
n_2_@DiC8	−15.3	479.1	*K*_d_	−16.2	700

^a^The effects of the counter ion PF_6_^−^ are included in the calculation. #*K* declares the equilibrium constant *K* with regard to Equation 1 and Figure 3.

Regarding the aforementioned deviations from experiment, the difference in the absolute Gibbs energies of association can be explained by the insufficient modeling of solvent effects. The solvent model assumes a uniform distribution of the two different solvents in the mixture. An explicit treatment of at least some solvent molecules would be desirable but is computationally not feasible at the required quantum mechanical level. A combined molecular mechanics/quantum mechanics treatment could be a solution to this problem in the future. Nevertheless, concerning the difference between Δ*G* in the gas phase and the experimental value, the solvent model that is used in this study yields a significant part of Δ*G*, but it cannot resolve details of the solvation effects.

**Figure 4:**
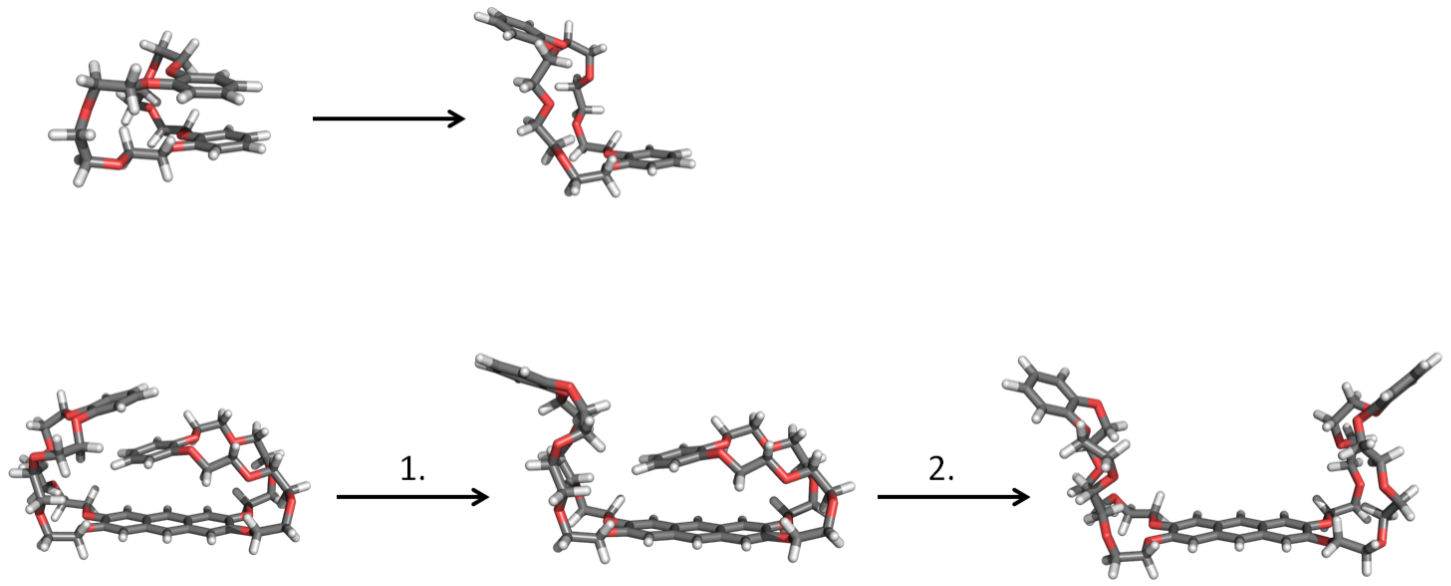
Optimized gas-phase structures for unfolding the monovalent (first row) and divalent (second row) host molecules. For the latter case a two-step process is found.

At the end of this discussion it is worth mentioning that the most stable structure of the host molecule changes from gas phase to solution. Both the monovalent and the divalent host have a folded ground state structure the in gas phase (Figure 4). The electronic energy Δ*E* that is needed for unfolding the monovalent host is 29.7 kJ/mol. This value increases up to 72.3 kJ/mol for fully unfolding the divalent host (52.6 kJ/mol for the first step and 19.6 kJ/mol for the second step). In solution (2.2:1 chloroform/acetonitrile, 298.15 K) the monovalent host is more stable in the unfolded form with Δ*G* being 8.2 kJ/mol lower than that of the folded form. The divalent host stays in the folded structure, and Δ*G* is 6.5 kJ/mol lower than that of the unfolded form.

## Conclusion

The Gibbs energies of association, including enthalpic and entropic temperature effects, solvent effects and the counter ions, have been determined for the divalent crown-8/ammonium pseudorotaxane with different linkers in the guest molecule. Additionally, a full double mutant cycle has been investigated in the same way. Our results agree with the experimental findings that the shortest investigated linker yields a strongly enhanced binding compared to the monovalent case due to the binding of the guest linker to the host linker. Our first principle calculations show clearly that this enhanced binding is due to electronic effects, namely the dispersion interaction of the two linkers. For the shortest linker this interaction results in a nearly ideal π–π stacking. For the two longer linkers ideal packing is not possible due to steric hindrance. These investigations proved that besides the primary binding sites in multivalent arrangements the interaction of the linkers can influence the binding process significantly. Therefore the term of non-innocent linkers introduced in [16] is well justified.
